# Comparative Evaluation of Preoperative Ultrasonography and Mammography in Breast Carcinoma: Correlation With Intraoperative and Histopathological Findings

**DOI:** 10.7759/cureus.115448

**Published:** 2026-08-30

**Authors:** Parmar Bhargav, Abhinav K Tiwari, Aditi Mittal, Raumil Parmar, Komal Khuman, Divyakant Barot, Vibhor Jain, Vivek Maurya, Dishant Parmar

**Affiliations:** 1 General Surgery, B.J. Medical College and Civil hospital, Ahmedabad, IND; 2 General Surgery, Gajara Raja Medical College, Gwalior, IND; 3 General Surgery, All India Institute of Medical Sciences, Rajkot, Rajkot, IND; 4 Obstetrics and Gynecology, Autonomous State Medical College, Shahjahanpur, IND; 5 Nephrology, Institute of Kidney Diseases and Research Centre, Ahmedabad, IND; 6 Medicine, B.J. Medical College and Civil Hospital, Ahmedbad, IND; 7 General Surgery, Autonomous State Medical College, Shahjahanpur, IND; 8 Community Health, Gujarat Medical Education and Research Society (GMERS) Medical College and Hospital, Ahmedabad, IND

**Keywords:** breast neoplasms, diagnostic imaging, histopathology, mammography, ultrasonography

## Abstract

Introduction: Accurate preoperative imaging is essential for optimal surgical planning in breast carcinoma. Mammography and ultrasonography are commonly used imaging modalities; however, their relative effectiveness in tumor size estimation and lymph node evaluation varies. This study aimed to compare the diagnostic role of preoperative ultrasonography and mammography in breast carcinoma and to correlate imaging findings with intraoperative and histopathological outcomes. Materials and methods: This prospective comparative observational study included 50 female patients with malignant breast disease undergoing elective surgery at a tertiary care teaching hospital. All patients underwent preoperative mammography and ultrasonography. Imaging findings were correlated with intraoperative observations and histopathological examination. Statistical analysis was performed using SPSS Statistics version 26.0 (IBM Corp. Released 2019. IBM SPSS Statistics for Windows, Version 26.0. Armonk, NY), with p < 0.05 considered statistically significant. Results: The mean age of patients was 51.38 ± 9.65 years. Ductal carcinoma was the most common histopathological subtype (62%, n = 31). Ultrasonography demonstrated a mean tumor size of 2.97 ± 0.46 cm, which was closer to the intraoperative tumor size (3.49 cm) than the mammographic estimate of 2.53 ± 0.41 cm. Both ultrasonographic and mammographic tumor-size estimates differed significantly from the intraoperative measurements (both p < 0.001). Ultrasonography showed a stronger association with intraoperative lymph node findings than mammography. Conclusions: Preoperative ultrasonography yielded a mean tumor-size estimate closer to the reference measurement than mammography and showed a stronger association with intraoperative lymph node findings. However, the combined use of both imaging modalities provides optimal diagnostic assessment and should be routinely employed in the preoperative evaluation of breast carcinoma.

## Introduction

Breast carcinoma is the most commonly diagnosed malignancy among women worldwide and remains a major cause of cancer-related mortality [1]. In India, its incidence continues to rise, particularly in urban areas, with delayed diagnosis remaining a significant challenge due to limited access to screening and diagnostics in many regions [2,3]. Accurate preoperative imaging is therefore essential for early diagnosis and appropriate surgical planning.

Breast carcinoma arises from the epithelial lining of the terminal duct-lobular unit, with invasive ductal carcinoma and invasive lobular carcinoma being the most common histological subtypes [4]. Patients may present with various symptoms, including mastalgia, nodularity, or a palpable breast lump, the latter requiring prompt and systematic evaluation [5].

The triple assessment approach (clinical examination, imaging, and tissue diagnosis) has become the standard for evaluating breast lesions, providing high diagnostic accuracy while reducing unnecessary surgical biopsies [6]. Mammography is widely accepted as the primary imaging modality for breast carcinoma screening and evaluation, particularly for detecting microcalcifications; however, its sensitivity is reduced in women with dense breast tissue due to tissue overlap [7,8].

Ultrasonography is a valuable adjunct to mammography, especially in dense breasts, as it enables improved lesion characterization and assessment of axillary lymph nodes [9,10]. Several studies have demonstrated that the combined use of mammography and ultrasonography improves diagnostic accuracy compared to either modality alone [11,12].

The Breast Imaging Reporting and Data System (BI-RADS) is a standardized classification developed by the American College of Radiology to categorize breast imaging findings based on the likelihood of malignancy. BI-RADS IV represents suspicious lesions requiring biopsy, while BI-RADS V denotes lesions highly suggestive of malignancy. In this study, BI-RADS IV and V lesions were analyzed for correlation with intraoperative and histopathological findings [13].

This study aimed to compare preoperative ultrasonography and mammography for tumor-size estimation and axillary lymph-node assessment in patients with breast carcinoma, using histopathological findings as the reference standard.

## Materials and methods

Study design and setting

This was a hospital-based, prospective, comparative observational study conducted in the Department of General Surgery at a tertiary care teaching hospital, Gajra Raja Medical College, Gwalior, India, over 12 months, from October 2020 to September 2021.

Study population

The study included 50 female patients diagnosed with breast carcinoma who were planned for elective surgical management during the study period. All eligible patients were informed about the study protocol, and written informed consent was obtained before enrollment.

Eligibility criteria

Female patients aged between 18 and 70 years with a diagnosis of malignant breast disease and planned for elective surgery were included in the study. Patients were excluded if they were younger than 18 years or older than 70 years, pregnant, had recurrent breast carcinoma, had received prior chemotherapy or radiotherapy, were unfit for anesthesia, had uncontrolled coagulopathy, were receiving anticoagulant therapy, refused participation, or were lost to follow-up before final evaluation.

Data collection and imaging evaluation

A structured questionnaire was developed by the authors specifically for this study. Clinical data were collected using a structured pro forma that included demographic details, clinical presentation, and physical examination findings. All patients underwent routine laboratory investigations along with preoperative breast imaging, including mammography and ultrasonography. Imaging findings were documented systematically for each patient.​​​​​​​​​​​​​​

Surgical procedure and histopathological correlation

Preoperative mammographic and ultrasonographic findings were compared with operative observations recorded during surgical intervention. All patients underwent modified radical mastectomy according to standard surgical guidelines. Resected specimens were sent for histopathological examination, which was considered the reference standard for assessing tumor size and axillary lymph node involvement. Operative findings were recorded separately as intraoperative observations.​​​​​​​

Statistical analysis

Data were entered into Excel (Microsoft Corp., Redmond, WA, USA) and analyzed using SPSS Statistics version 26.0 (IBM Corp. Released 2019. IBM SPSS Statistics for Windows, Version 26.0. Armonk, NY). Continuous variables were expressed as mean and standard deviation, and categorical variables were presented as frequencies and percentages. Because tumor-size measurements were obtained from the same patients by ultrasonography, mammography, and histopathological assessment, the comparisons represented paired observations. Statistical tests were interpreted accordingly. For lymph-node assessment, histopathological findings were considered the reference standard. A p-value of <0.05 was considered statistically significant.​​​​​​​

Ethics statement

Approval for the study was obtained from the Institutional Ethics Committee of Gajra Raja Medical College prior to commencement (approval number: 36/IEC-GRMC/2019). Written informed consent was obtained from all human participants before enrollment. Patient confidentiality was strictly maintained, and no identifiable personal information was included in the study.

## Results

The age distribution of patients with breast carcinoma showed that the majority were in the 51-60-year age group, followed by those aged 41-50 years (Figure 1).

**Figure 1:**
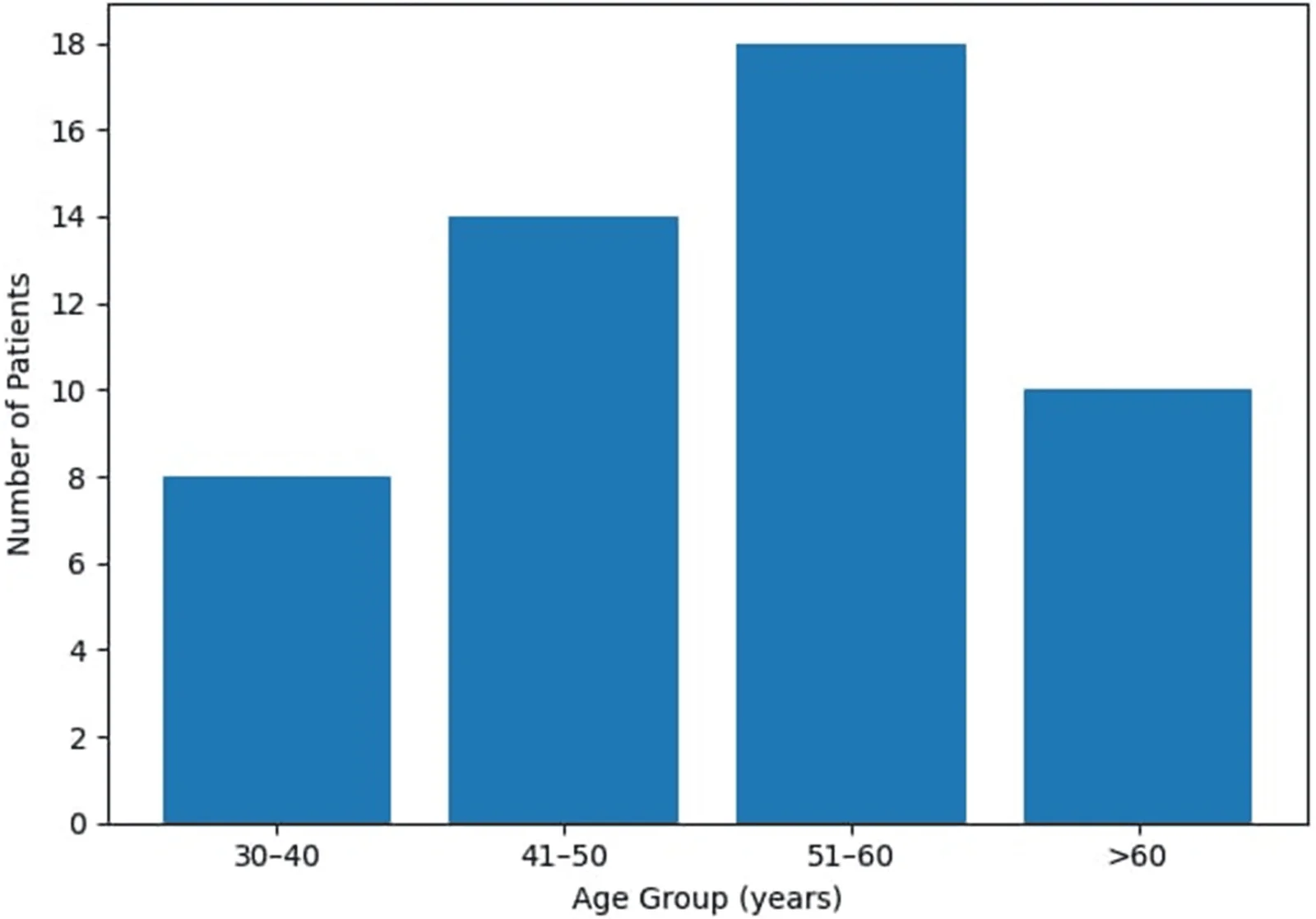
Age distribution of study participants Bar diagram showing the age distribution of patients with breast carcinoma included in the study. The majority of patients were in the 51-60-year age group, followed by the 41-50-year age group, with most participants being in the peri- and postmenopausal age groups.

A total of 50 female patients with malignant breast disease were included in the study. The mean age was 51.38 ± 9.65 years (range: 34-68 years). Invasive ductal carcinoma was the most common histopathological subtype, observed in 62% (n = 31) of cases, followed by invasive lobular carcinoma in 38% (n = 19). All patients underwent modified radical mastectomy (Table 1).

**Table 1:** Ultrasonographic findings Continuous variables are presented as mean ± SD, while categorical variables are presented as frequencies and percentages. SD: standard deviation

Parameter	Value
Mean tumor size (cm) ± SD	2.97 ± 0.46
Tumor size >2 cm	45 (90%)
Lymph nodes detected	34 (68%)
Calcification present	31 (62%)
BI-RADS V	33 (66%)

On ultrasonography, the mean tumor size was 2.97 ± 0.46 cm, with 90% (n = 45) of tumors measuring >2 cm. Axillary lymph nodes were detected in 68% (n = 34) of patients, while calcifications were present in 62% (n = 31). The majority of lesions (66%, n = 33) were categorized as BI-RADS V (Table 2).

**Table 2:** Mammography findings Continuous variables are presented as mean ± SD, while categorical variables are presented as frequencies and percentages. SD: standard deviation, BI-RADS V: Breast Imaging Reporting and Data System category V

Parameter	Value
Mean tumor size (cm) ± SD	2.53 ± 0.41
Tumor size >2 cm	41 (82%)
Lymph nodes detected	36 (72%)
Calcification present	35 (70%)
BI-RADS V	38 (76%)

Mammography showed a mean tumor size of 2.53 ± 0.41 cm, with 82% (n = 41) of tumors measuring >2 cm. Axillary lymph node detection and calcifications were identified in 72% (n = 36) and 70% (n = 35) of cases, respectively. A higher proportion of lesions were classified as BI-RADS V (76%, n = 38) compared to ultrasonography (Table 3).

**Table 3:** Comparison of preoperative imaging findings and intraoperative findings

Parameter	Ultrasonography (n = 50)	Mammography (n = 50)	Intraoperative (n = 50)
Mean tumor size (cm)	2.97	2.53	3.49
Lymph node detection (%)	68%	72%	94%
Vascularity present (%)	-	-	76%

Comparative analysis of BI-RADS categories on ultrasonography and mammography revealed greater radiological suspicion on mammography (Figure 2).

**Figure 2:**
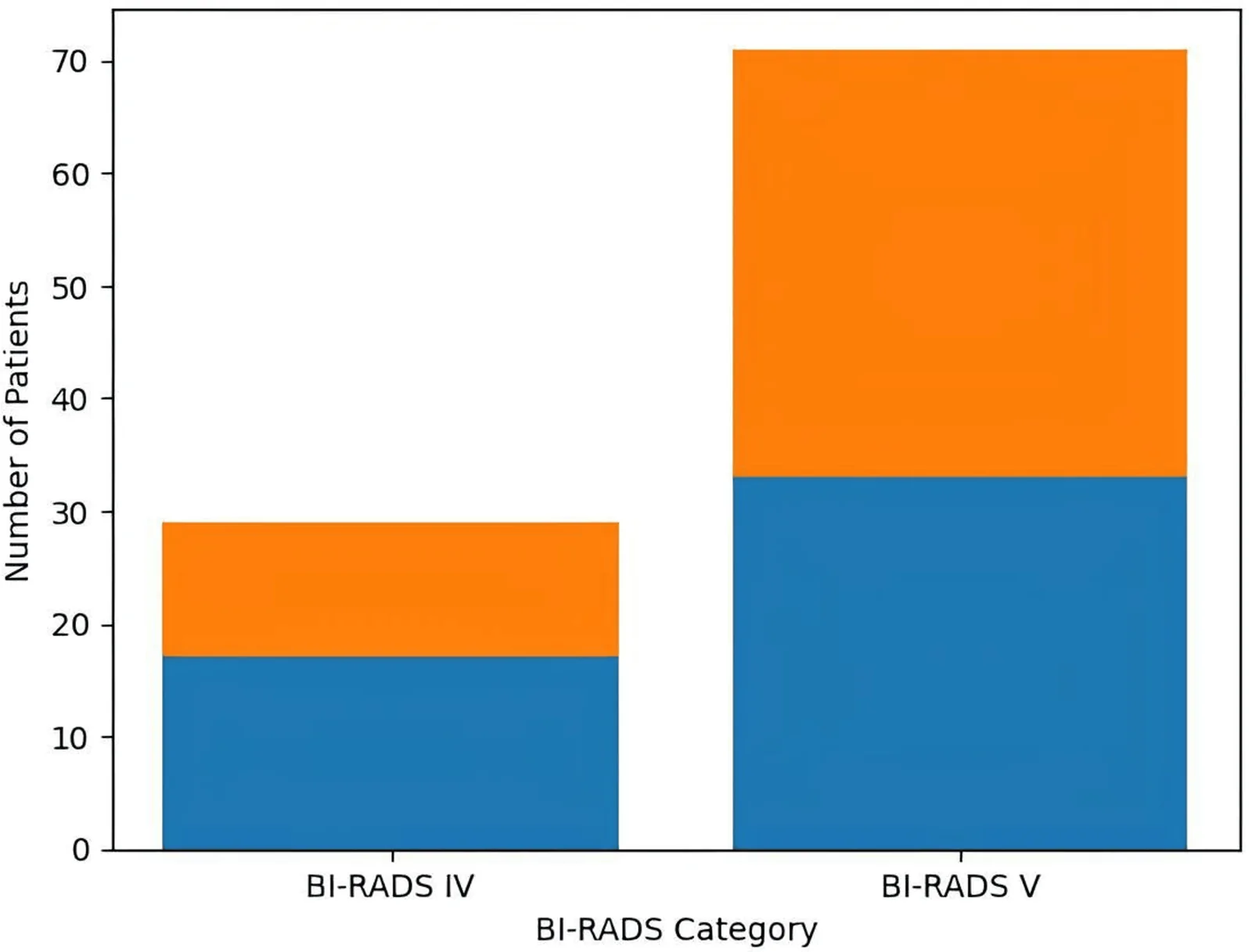
Comparative distribution of BI-RADS categories on ultrasonography and mammography Comparative bar chart showing BI-RADS categorization of malignant breast lesions on ultrasonography and mammography. Mammography classified a higher proportion of lesions as BI-RADS V than ultrasonography, indicating greater radiological suspicion on mammographic evaluation. Blue bars indicate ultrasonographic findings and orange bars indicate mammography findings. BI-RADS: Breast Imaging Reporting and Data System

Histopathological assessment demonstrated a mean tumor size of 3.49 cm, which was higher than the mean tumor size measured by ultrasonography and mammography. Ultrasonography measured a mean tumor size of 2.97 cm, which was closer to the intraoperative measurement than mammography (2.53 cm). Axillary lymph nodes were detected intraoperatively in 94% of cases, whereas ultrasonography and mammography detected lymph nodes in 68% and 72% of cases, respectively. Tumor vascularity was observed intraoperatively in 76% of patients (Table 4).

**Table 4:** Comparison of mean tumor-size measurements with histopathological assessment

Modality	Mean predicted size (cm)	Mean intraoperative size (cm)	Difference between means (cm)
Ultrasonography	2.97	3.49	-0.52
Mammography	2.53	3.49	-0.96

The ultrasonographic mean tumor-size estimate was closer to the intraoperative mean than the mammographic estimate (Figure 3).

**Figure 3:**
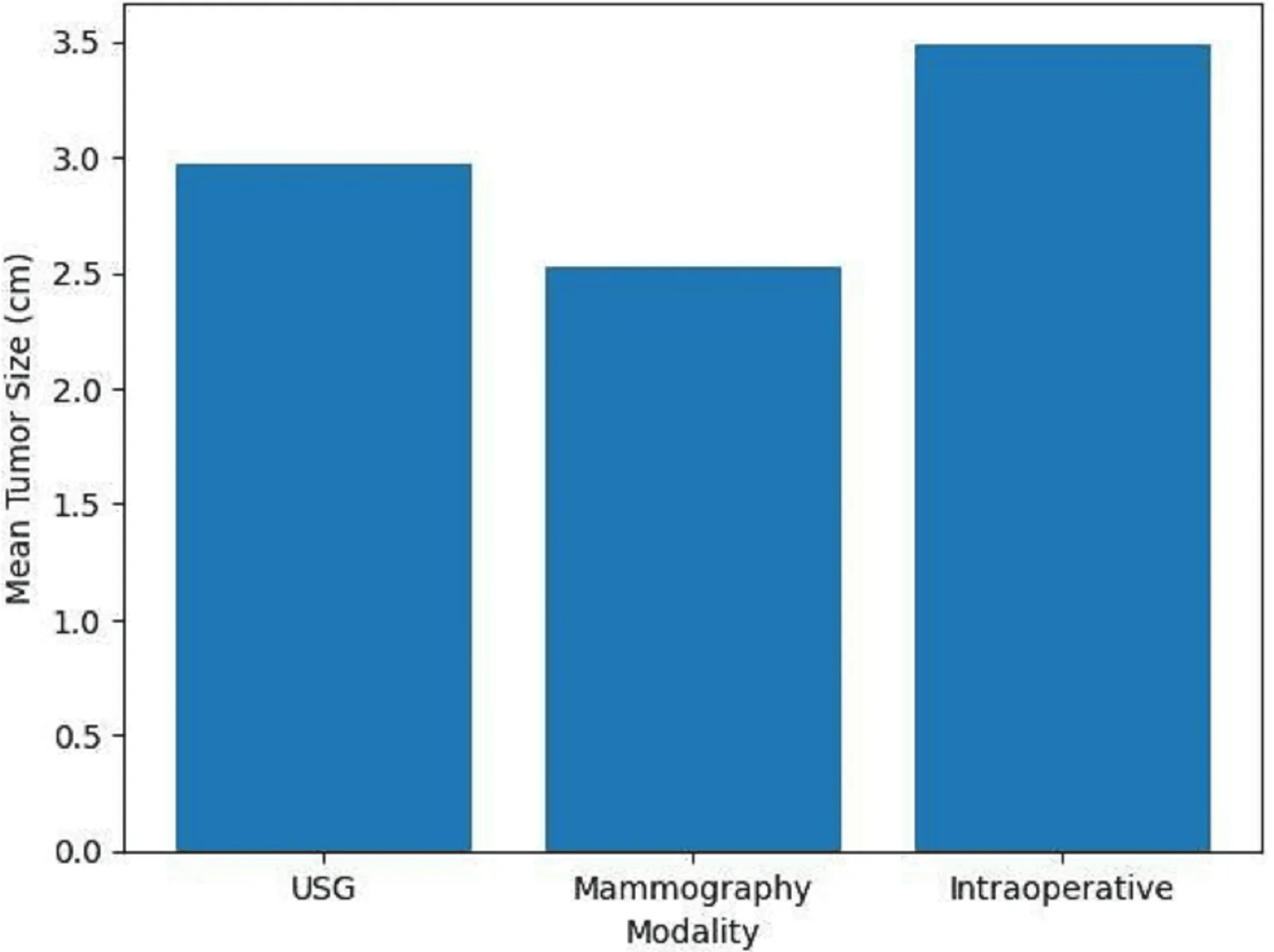
Comparison of mean tumor size measurements obtained by ultrasonography, mammography, and intraoperative assessment Bar chart comparing mean tumor size measured by ultrasonography, mammography, and intraoperative assessment. Ultrasonographic measurements showed greater agreement with intraoperative tumor size than mammography.

Lymph node detection rates varied across assessment modalities. Intraoperative examination demonstrated the highest lymph node detection rate, followed by mammography and ultrasonography. Ultrasonographic lymph node detection showed a statistically significant association with operative findings, whereas mammographic detection did not (Figure 4).

**Figure 4:**
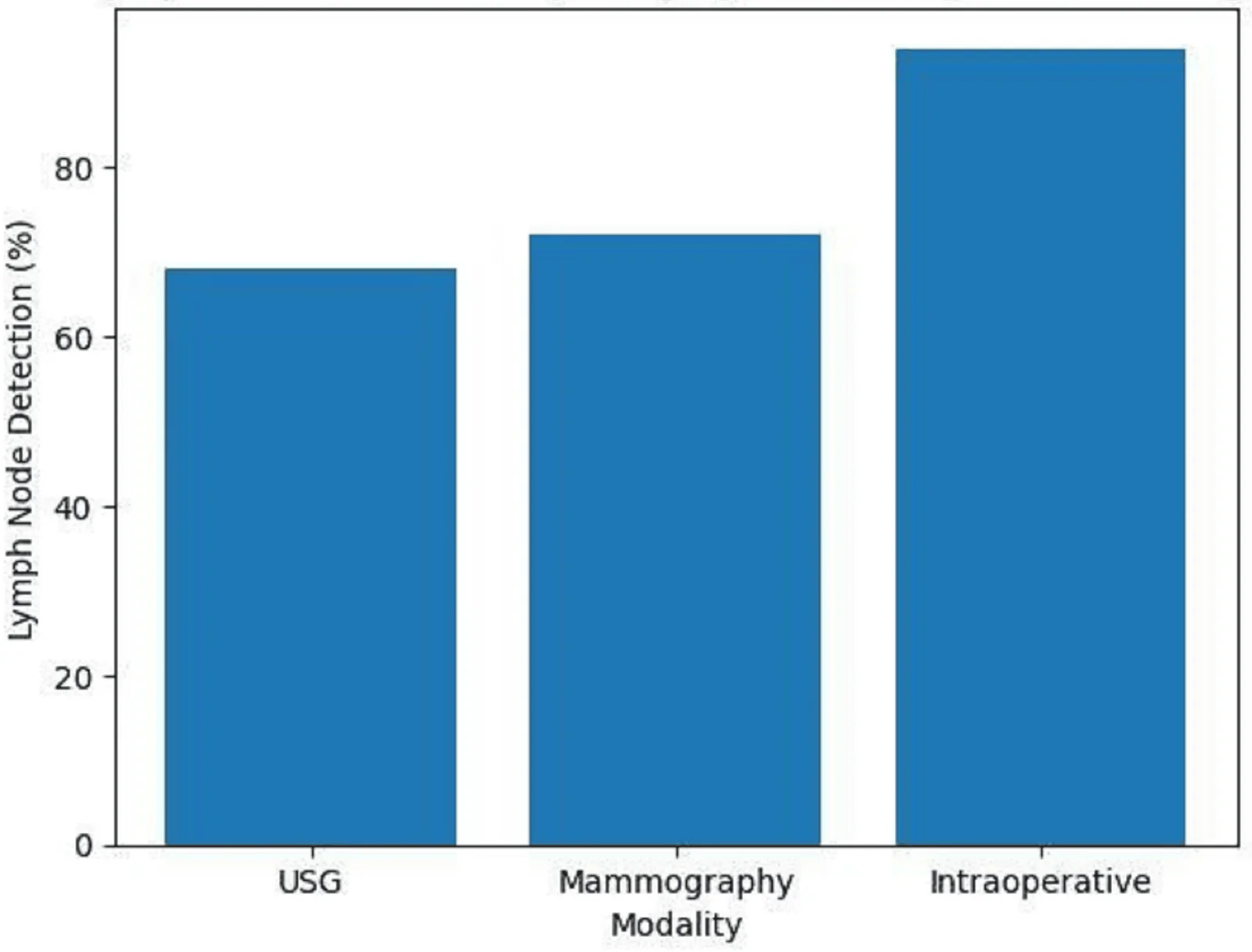
Lymph node detection by imaging modalities and intraoperative findings Bar diagram illustrating lymph node detection rates by ultrasonography, mammography, and intraoperative examination. Intraoperative assessment demonstrated the highest lymph node detection rate, while ultrasonographic lymph node detection showed an association with operative findings.

Ultrasonography demonstrated a mean tumor size of 2.97 cm, compared with a histopathological mean of 3.49 cm, yielding a difference of −0.52 cm. Mammography showed a mean tumor size of 2.53 cm, corresponding to a difference of −0.96 cm from the histopathological mean. Thus, the ultrasonographic mean was numerically closer to the histopathological mean than was the mammographic mean. These values are presented as descriptive comparisons rather than diagnostic accuracy estimates (Table 4).

Table 5 compares imaging findings with histopathological and operative findings. Ultrasonography demonstrated a statistically significant difference in tumor size compared with histopathology (t = −8.39, df = 49, p < 0.001). Mammography also demonstrated a statistically significant difference from histopathological tumor size (t = −12.32, df = 49, p < 0.001). The smaller magnitude of the difference in mean measurements observed with ultrasonography suggests that its mean tumor-size estimate was closer to the histopathological mean than that of mammography. For axillary lymph-node detection, ultrasonography showed a borderline statistically significant association with operative findings (χ² = 3.86, df = 1, p = 0.049). Because the observations were obtained from the same patients and operative lymph node detection does not establish metastatic involvement, this finding was interpreted with caution. Mammography did not demonstrate a statistically significant association with intraoperative lymph node status (p > 0.05). Overall, ultrasonography yielded a mean tumor size estimate closer to that of histopathological assessment than mammography. However, the present analysis should be interpreted as a comparison of mean measurements rather than a formal agreement analysis.

**Table 5:** Comparison of imaging findings with histopathological and operative findings

Comparison	Result	p-value
Ultrasonographic vs intraoperative tumor size	t = -8.39, df = 49	<0.001
Mammographic vs intraoperative tumor size	t = -12.32, df = 49	<0.001
Ultrasonographic lymph node detection vs operative findings	χ² = 3.86 , df = 1	0.049
Mammographic lymph node detection vs operative findings	Not statistically significant	>0.05

Representative ultrasonographic and mammographic appearances of breast carcinoma (BI-RADS V) are illustrated in Figure 5.

**Figure 5:**
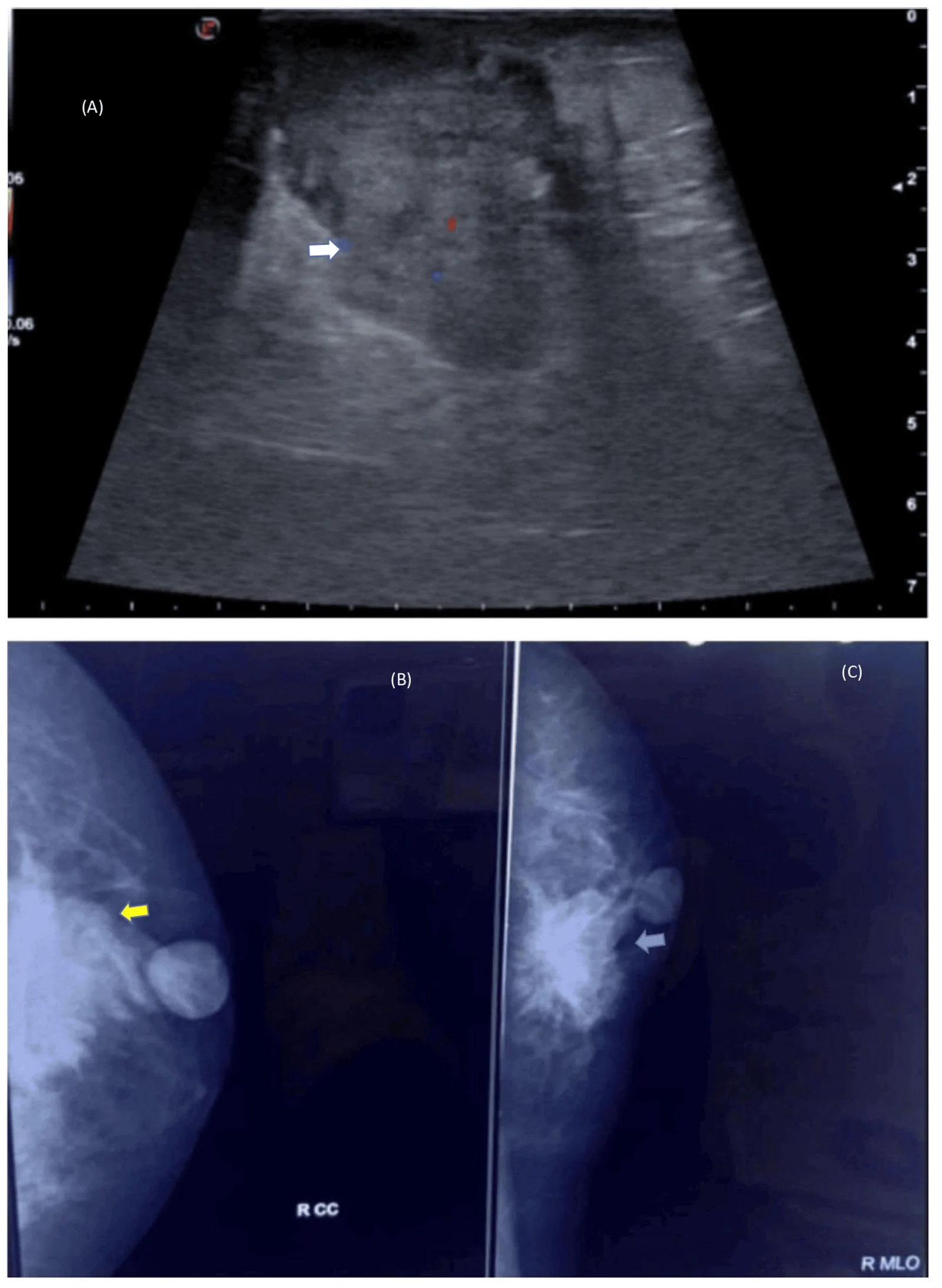
Mammographic and ultrasonographic appearance of right breast carcinoma (case illustration) Multimodality imaging of the right breast lesion. (A) Ultrasonography image showing an ill-defined heterogeneous lesion in the subareolar region of the right breast (white arrow). (B) RCC mammographic view demonstrating a well-defined oval-shaped radiopaque lesion in the subareolar region of the right breast (yellow arrow). (C) RMLO mammographic view showing the corresponding oval-shaped radiopaque lesion in the subareolar region of the right breast (blue arrow). RCC: right craniocaudal, RMLO: right mediolateral oblique Image Credit: Original images acquired by the authors during routine diagnostic evaluation at the study institution.

## Discussion

In the present study, ultrasonography yielded a mean tumor size estimate closer to that of histopathological assessment than mammography, indicating a smaller difference from the histopathological mean [8,9]. This finding is consistent with prior studies that have reported improved concordance between ultrasonographic and pathological tumor measurements, particularly in patients with dense breast tissue [13,14]. Accurate preoperative assessment of tumor size is critical for surgical planning, selection of breast-conserving surgery, and achieving clear resection margins, underscoring the clinical relevance of these findings [15].

Mammography yielded a lower mean tumor-size estimate than the reference measurement in the present study, a limitation widely reported in the literature. The observed underestimation may be related to factors such as tissue superimposition and reduced lesion conspicuity described in previous studies [16,17]. However, breast density was not systematically recorded in the present cohort; therefore, its specific contribution to the observed difference could not be evaluated. Although mammography remains the cornerstone of breast carcinoma screening due to its high sensitivity for lesion detection, several studies have highlighted its limited accuracy in determining true tumor dimensions [13,18]. Our findings further support these observations and reinforce the need for adjunct imaging modalities when precise assessment of tumor size is required [19].

Despite mammography classifying a greater proportion of lesions as BI-RADS V, this did not translate into improved accuracy for tumor size measurement. Similar discrepancies between BI-RADS categorization and actual tumor size have been described in previous studies, suggesting that while BI-RADS effectively stratifies malignancy risk, it may not reliably predict tumor extent, as BI-RADS assessment focuses on lesion suspicion rather than dimensional accuracy [18]. In contrast, ultrasonography allows real-time imaging, improved lesion-margin delineation, and better differentiation between tumor tissue and surrounding parenchyma, particularly in dense breasts, thereby facilitating tumor-size assessment [9,20].

Axillary lymph node assessment in the present study demonstrated the highest intraoperative detection rate, followed by mammography and ultrasonography. Ultrasonography detected lymph nodes in 68% of patients, compared with 72% for mammography. However, imaging-based or operative lymph node detection does not establish metastatic involvement; therefore, histopathological examination remains the appropriate reference standard for diagnosing nodal metastasis [21]. This is consistent with existing evidence indicating that ultrasonography is superior in evaluating axillary nodal morphology, including cortical thickening, eccentric cortical hypertrophy, and loss of the fatty hilum, features strongly associated with metastatic involvement [22,23]. Mammography, by contrast, has limited sensitivity for assessing axillary lymph nodes and is largely restricted to detecting gross nodal enlargement or calcifications [23].

The representative case included in this study further illustrates the complementary roles of mammography and ultrasonography in the preoperative evaluation of breast carcinoma, with imaging findings corroborated by cytology and histopathology. However, the findings should be interpreted in light of the study’s sample size and single-center design. Taken together, the present findings align with existing literature and support the combined use of mammography and ultrasonography to improve diagnostic accuracy, optimize surgical planning, and enhance preoperative staging in patients with breast carcinoma [24].

Limitations

The study was limited by its relatively small sample size and single-center design. Inter-observer variability and formal blinding were not assessed, and breast density was not systematically recorded. These factors should be considered when interpreting the findings. Further multicenter studies with larger sample sizes are recommended.

## Conclusions

In this prospective comparative study of 50 patients with breast carcinoma, ultrasonography yielded a mean tumor-size estimate closer to the reference measurement than mammography and showed a stronger association with intraoperative lymph node findings. Mammography remained valuable for lesion detection and classification, providing complementary diagnostic information. Therefore, ultrasonography and mammography should be regarded as complementary rather than mutually exclusive modalities, and their combined use may provide a more comprehensive preoperative assessment to guide surgical management in patients with breast carcinoma.
